# Novel and Efficient Quantitative Posterior-Circulation-Structure-Based Scale via Noncontrast CT to Predict Ischemic Stroke Prognosis: A Retrospective Study

**DOI:** 10.3390/jpm12020138

**Published:** 2022-01-20

**Authors:** Wen-Hui Fang, Ying-Chu Chen, Ming-Chen Tsai, Pi-Shao Ko, Ding-Lian Wang, Sui-Lung Su

**Affiliations:** 1 Department of Family and Community Medicine, Tri-Service General Hospital, National Defense Medical Center, Taipei 11490, Taiwan; rumaf.fang@gmail.com; 2 School of Public Health, National Defense Medical Center, Taipei 11490, Taiwan; joanna098501072@gmail.com (Y.-C.C.); kimirrarike@gmail.com (P.-S.K.); dl.wang19971224@gmail.com (D.-L.W.); 3 Department of Neurology, Tri-Service General Hospital, National Defense Medical Center, Taipei 11490, Taiwan; immingchentsaiha@gmail.com; 4 Graduate Institute of Life Sciences, National Defense Medical Center, Taipei 11490, Taiwan

**Keywords:** posterior circulation ischemic stroke, deep learning, noncontrast CT, pc-ASPECTS

## Abstract

(1) Background: Posterior circulation ischemic stroke has high mortality and disability rates and requires an early prediction prognosis to provide the basis for an interventional approach. Current quantitative measures are only able to accurately assess the prognosis of patients using magnetic resonance imaging (MRI). However, it is difficult to obtain MRI images in critically urgent cases. Therefore, the development of a noncontrast CT-based rapid-assist tool is needed to enhance the value of the clinical application. (2) Objective: This study aimed to develop an auxiliary-annotating noncontrast CT-efficient tool, which is based on a deep learning model, to provide a quantitative scale and the prognosis of posterior circulation ischemic stroke patients. (3) Methods: A total of 31 patients with posterior circulation ischemic stroke, diagnosed in the stroke registry at the Tri-Service General Hospital from November 2019 to July 2020, were included in the study, with a total of 578 CT images collected from noncontrast CT and MRI that were ≤ 3 days apart. A 5-fold cross validation was used to develop an image segmentation model to identify nine posterior circulation structures, and intersection over union (IoU) was used to assess the ability of the model to identify each structure. A quantitative score was integrated to assess the importance of the proportion of ischemic lesions in each posterior circulation structure, and the ROC curve was compared with the semiquantitative score for prognostic power. The prognoses of the patients were defined into two groups of 18 patients. An mRS score of 0–2 at discharge was defined as a good prognosis, while an mRS score of 3–6 was deemed to be a poor prognosis. (4) Results: The performance of the image segmentation model for identifying the nine posterior circulation structures in noncontrast CT images was evaluated. The IoU of the left cerebellum was 0.78, the IoU of the right cerebellum was 0.79, the IoU of the left occipital lobe was 0.74, the IoU of the right occipital lobe was 0.68, the IoU of the left thalamus was 0.73, the IoU of the right thalamus was 0.75, the IoU of the medulla oblongata was 0.82, and the IoU of the midbrain was 0.83. The prognostic AUC of posterior circulation patients predicted using a quantitative integrated score was 0.74, which was significantly higher than that of the pc-ASPECTS (AUC = 0.63, *p* = 0.035), with a sensitivity of 0.67 and a specificity of 0.72. (5) Conclusions: In this study, a deep learning model was used to develop a noncontrast CT-based quantitative integrated score tool, which is an effective tool for clinicians to assess the prognosis of posterior circulation ischemic stroke.

## 1. Introduction

There are approximately 2.93 million cases of posterior circulation ischemic stroke around the globe each year [1,2]. The death and disability risks are high, and an accurate prognosis assessment can provide a reference for physicians and families in the face of high-risk treatment measures. Therefore, a quantitative assessment of patient prognosis is required for a better understanding of the prognosis of patients with posterior circulation ischemic stroke, after the intervention.

An efficient triage of patients for additional imaging diagnostics, adequate therapy regimes, and initial outcome prediction requires the detection of early ischemic changes in early hyperacute noncontrast computed tomography (noncontrast CT) scans [3], which are commonly used in the diagnosis of acute ischemic stroke. Noncontrast CT can provide useful cerebral information in a short amount of time [4]. If noncontrast CT can be used as the basis of interpretation, it can provide an early reference for intervention. The current quantitative tools are mostly based on magnetic resonance imaging (MRI), which is the most sensitive tool for the diagnosis of ischemic stroke; however, it can involve a longer time for the examination, a more restrictive environment, higher costs, limited availability and accessibility (especially in emergencies), and patient intolerance or incompatibility [5]. Therefore, for acute patients, it is difficult to obtain MRI images for physician evaluation.

A quantitative assessment of patients with ischemic stroke was proven to be more accurate in predicting prognosis [6], and there is better agreement across studies in defining poor prognoses in terms of volume [7] because of the limitations of semiquantitative assessment. Ischemic lesions involving structural areas of the brain are scored equally, regardless of their volume and proportion in the structure, and only the number of different structures involved in the ischemic lesion can be assessed. Currently, semiquantitative methods are used owing to the urgency of clinical evaluation. However, the accuracy of a CT-based assessment of the prognosis of patients with posterior circulation ischemic stroke is subpar [8], and even the posterior circulation-Acute Stroke Prognosis Early CT Score (pc-ASPECTS: a semiquantitative scale, which draws on the concept of the ASPECTS, and scores the eight locations of the posterior circulation of the brain), calculated by noncontrast CT, may not be able to predict the prognosis of patients. Even the advanced imaging perfusion CT (CTP) also has limited diagnostic sensitivity and suboptimal prognosis [9].

The prognostic assessment of patients with posterior circulation ischemic stroke is urgent and crucial, and it requires the quantitative and accurate assessment of noncontrast CT scans and shortened assessment times in order to provide an early reference for intervention. Therefore, there is a need to develop a rapid quantitative tool to effectively assess the prognosis of patients with posterior circulation ischemic stroke.

## 2. Materials and Methods

### 2.1. Ethical Issues

The Institutional Review Board’s Tri-Service General Hospital (TSGH), a medical teaching hospital of the National Defense Medical Center in Taipei, Taiwan, approved this retrospective study and waived the need for informed consent (C202005119).

### 2.2. Data Collection

**Figure 1:**
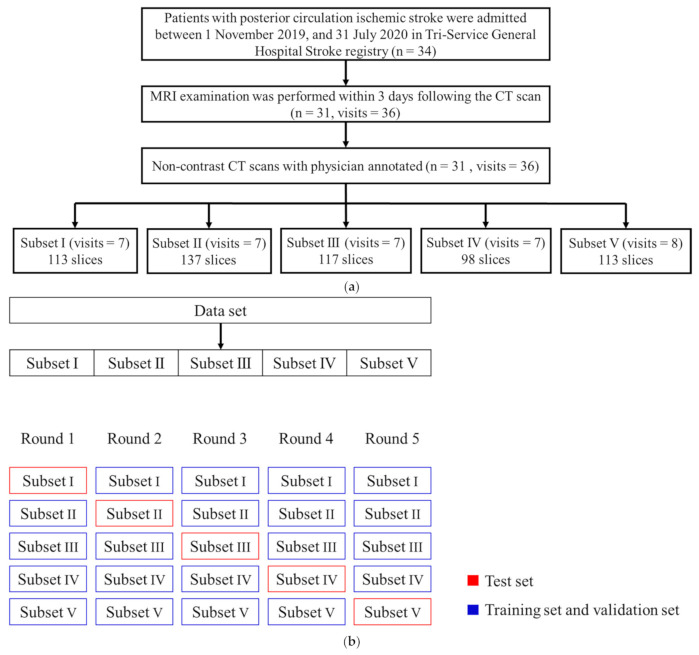
Flow chart of case admission and 5-fold cross-validation method. (**a**) Flow chart of case admission: The research subjects were patients with a confirmed diagnosis of posterior circulation ischemic stroke in the stroke registry at TSGH between 1 November 2019 and 31 July 2020, and those with an interval of fewer than three days between CT and MRI exams were selected, and their images were annotated by neurologists. A total of 31 patients participated in the research, and there was a total of 36 visits, taking into account recurrences and repeated visits during the study period. The subjects were divided into five subsets to conduct the 5-fold cross-validation analysis. (**b**) The 5-fold cross-validation method: after dividing the admitted subjects into five subsets, each subset would be used as the test set in turn, and the model performance would be evaluated by predicting the results of the test sets in five rounds of training and testing. Finally, the model performance was evaluated using the prediction results of the test sets. The subsets in blue were used as training and validation sets to construct the model, and the subsets in red were used as test sets for the model prediction.

We performed a retrospective study. The admission flow chart for patients with posterior circulation ischemic stroke from November 2019 to July 2020 in the stroke registry at TSGH is shown in Figure 1a. The inclusion criteria of this study were patients diagnosed with posterior circulation ischemic stroke, and the modified Rankin Scale (mRS) had to be available as patient data at the time of discharge. Those who did not complete both CT and MRI examinations (≤3 days apart) were excluded. There was a total of 31 patients who met the admission criteria and who were annotated by a neurologist; some of them were readmitted for recurrent strokes during the study period. The data included a total of 36 visits.

In this study, noncontrast CT and diffusion-weighted images (DWI) were acquired and annotated by a neurologist using the self-developed software, Xannotation, to depict nine posterior circulation structures in noncontrast CT images: the left cerebellum, the right cerebellum, the left lateral occipital lobe, the right lateral occipital lobe, the left lateral thalamus, the right lateral thalamus, the medulla oblongata, the midbrain, the pons, as well as the extent of the ischemic lesion. During the annotation process, the corresponding MRI images were also referenced in parallel. A total of 578 noncontrast CT images, with a 512 × 512-pixel size, were annotated for the 36 visits/cases. The data collected from the study were randomly divided into five subsets according to the number of visits, and each subset was used as a test set in turn to perform the 5-fold cross validation, as shown in Figure 1b.

### 2.3. Deep Learning Model Development and Training

Before the construction of the deep learning model, the head CT images in the dataset were preprocessed. Pixel value normalization was performed before the images were input into the model (512 × 512-pixel size). The images were subjected to online image augmentation techniques, such as random cropping, from a 512 × 512-pixel size into a 480 × 480-pixel size, or random scaling (10%) and the processing of the image to a 480 × 480-pixel size, while training the models.

The segmentation model uses the ResNet-18 architecture as the model’s encoder and performs transfer learning, while the decoder architecture is SegNet [7,8,9]. Adam is an effective variant of an optimization algorithm called “stochastic gradient descent”, which iteratively applies updates to the parameters in order to minimize the loss during training [8]. We trained the networks with minibatches of a size of 4, and used an initial learning rate of 0.001, which was decayed by a factor of 10 each time the loss on the tuning set plateaued after an epoch. The L2 regularization (weight decay, wd) was 0.0001. In order to prevent the networks from overfitting, early stopping was performed by saving the network after every epoch, and by choosing the saved network with the lowest loss on the tuning set. The samples were divided into five subsets according to Figure 1a, and were evaluated using 5-fold cross validation, as shown in Figure 1b, where four subsets were used as training sets, and some data were randomly used as a tuning set. The one subset left was used as the test set to evaluate the model identification performance. Each subset was used as the test set in turn, and the other subsets were used for training and parameter tuning [10].

### 2.4. Calculation of the Prediction Score

Using the noncontrast CT images annotated by the neurologist, the volumes of the nine posterior circulation structures and the volumes of the ischemic lesions were calculated for each patient, and the proportion of lesions in each posterior circulation structure and the proportion of ischemic lesions in other regions were converted to a total of 10 parameters. The samples were divided into five subsets, as shown in Figure 1a, and were evaluated using 5-fold cross validation, as shown in Figure 1b, with four subsets used as modeling data, and one subset as the test set. The quantitative integrated scores of this subset of patients were calculated according to the model parameter weights, and the prognoses of the patients were predicted to be favorable or not, and the quantitative integrated score of each subset was calculated five times. Patients with a modified Rankin Scale (mRS) score of 0–2 were defined as having a good prognosis, and those with a score of 3–6 were defined as having a poor prognosis.

The posterior circulation-Acute Stroke Prognosis Early CT Score (pc-ASPECTS) is calculated as follows: The Pc-ASPECTS allots the posterior circulation 10 points. One point each is subtracted for ischemic lesions on noncontrast CT in the left or right thalamus, cerebellum, or posterior cerebral artery (PCA) territory, and 2 points each are subtracted for ischemic lesions on noncontrast CT in any part of the midbrain or pons. A pc-ASPECTS of 10 indicates the absence of visible posterior circulation ischemia, and a score of 0 indicates ischemic lesions in all pc-ASPECTS territories [6]. The calculation of the quantitative integrated scores and the pc-ASPECTS was based on the areas annotated on the noncontrast CT images by the neurologist with reference to the MRI images.

### 2.5. Statistical Analysis and Evaluation on the Validation Set

The intersection over union (IoU) is the similarity between annotated pixels and predicted pixels, defined as the size of the intersection divided by the size of the union of the sample sets. The performance of the image segmentation model was evaluated using the IoU metric [11]. The prognostic ability of the quantitative integrated scores was evaluated by sensitivity, specificity, the ROC curve, and the AUC, and was compared with the semiquantitative scores. The difference in the area under the two curves was examined by the DeLong test, with *p* < 0.05 as the criterion for achieving statistically significant differences.

R (version 3.4.2) was used for programming, including data processing, statistical analysis, and model training; MXNet (version 1.3.1) was used for constructing the convolutional neural network model, and pROC (version 1.13.0) was used for calculating the area under the ROC curve.

## 3. Results

The basic demographic distribution of the subjects included in the study is described in Table 1, with 18 good and 18 poor prognoses: for female patient visits, there were 5 (27.8%) good prognoses and 11 (61.1%) poor prognoses, suggesting significantly worse prognoses than the male patient visits (*p* = 0.044). In terms of age, the mean age of the poor prognosis group was significantly higher than that of the good prognosis group (*p* = 0.007). The semiquantitative assessment using the pc-ASPECTS, however, did not show a significant difference between the two groups (*p* = 0.164). The gender- and age-stratified analyses also showed similar results (Tables S1 and S2).

**Figure 2:**
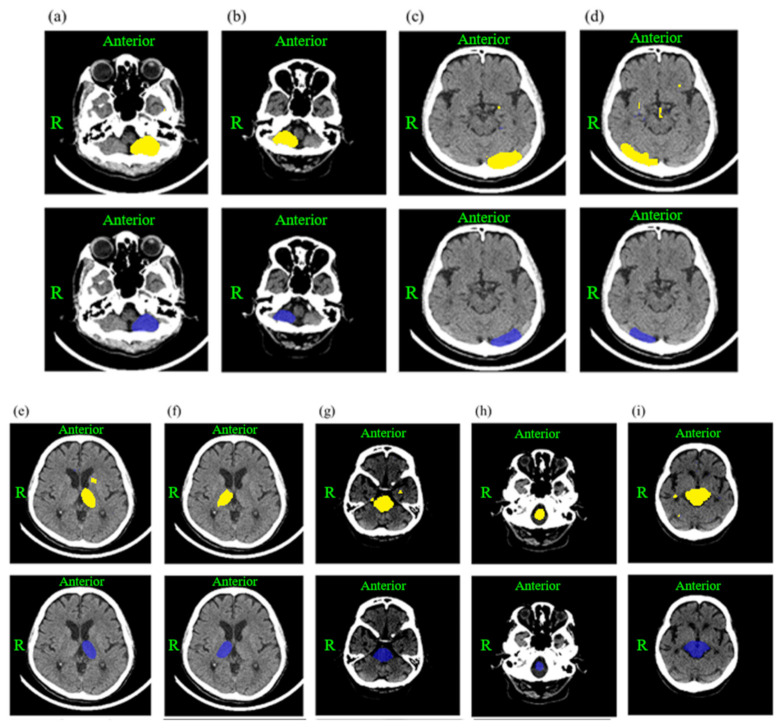
Schematic diagram comparing the structural range predictions by the image segmentation model and physician annotation. The upper half shows the structural regions according to the model prediction results of the model’s prediction of the structural area, which are presented in yellow, and the lower half shows the structural regions annotated by the physician, which are presented in blue. There are a total of nine cerebral structures for posterior circulation: (**a**) left cerebellum; (**b**) right cerebellum; (**c**) left occipital lobe; (**d**) right occipital lobe; (**e**) left thalamus; (**f**) right thalamus; (**g**) medulla oblongata; (**h**) midbrain; and (**i**) pons.

The prediction results of the image segmentation model in this study were compared with the structure range annotated by the physician. Figure 2 is a schematic diagram showing: (a) the left cerebellum; (b) the right cerebellum; (c) the left occipital lobe; (d) the right occipital lobe; (e) the left thalamus; (f) the right thalamus; (g) the medulla oblongata; (h) the midbrain; and (i) the pons, totaling nine cerebral structures for posterior circulation. The upper half is the model-predicted structural area results, which are presented in yellow, and the lower half is the physician-annotated structural area results, which are presented in blue. The performance of the circulation structure after the image segmentation model recognition is described in Table 2. The average IoU values of the nine posterior circulation structures were: 0.78 for the left cerebellum; 0.79 for the right cerebellum; 0.74 for the left occipital lobe; 0.68 for the right occipital lobe; 0.73 for the left thalamus; 0.75 for the right thalamus; 0.82 for the medulla oblongata; 0.83 for the midbrain; and 0.75 for the pons.

**Figure 3:**
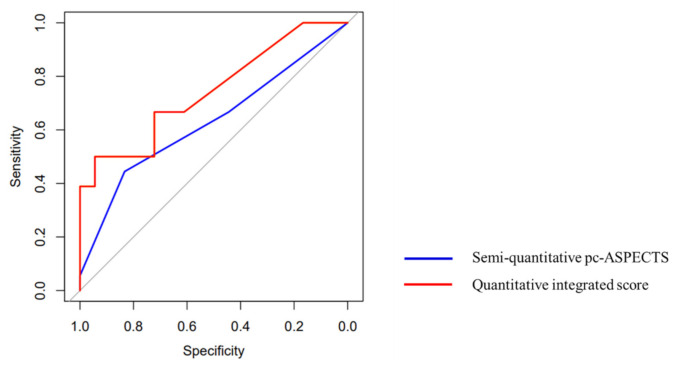
ROC curves of quantitatively and semiquantitatively calculated scores for prognosis prediction. The AUC for prognosis prediction by integrated quantitative score was 0.74, with a sensitivity of 0.67, and a specificity of 0.72. The AUC for prognosis prediction by the pc-ASPECTS calculated semiquantitatively, with a sensitivity of 0.67 and a specificity of 0.44, was 0.63. The results of the DeLong test show that the ROC for predicting prognosis using the quantitative integrated score was significantly better (*p* = 0.035).

As shown in Figure 3, the AUC for the prognosis prediction by an integrated quantitative score was 0.74, which was significantly different from the AUC of 0.63 for prognosis prediction by the pc-ASPECTS, calculated in a semiquantitative manner (*p* = 0.035), indicating that the ability of the quantitative integrated score in prognosis prediction was more accurate, with a sensitivity of 0.67, and a specificity of 0.72.

## 4. Discussion

This model can effectively quantify each structure of the posterior circulation and the ischemic lesion diagnosed by the physician, and automatically calculate the patient’s quantitative integrated score for the physician’s reference of the prognosis of patients with posterior circulation ischemic stroke. The predictive ability of the quantitative integrated score is superior to the semiquantitative method of the pc-ASPECTS.

Because of the apparent neurological-related symptoms in patients with anterior circulation ischemic stroke, it is relatively easy to define the time of the onset of stroke symptoms, and the National Institutes of Health Stroke Scale (NIHSS) can be used to effectively assess the severity of patients with anterior circulation. In addition, a sophisticated automated imaging system has been developed, which can automatically read the ASPECTS and the area of infarction [12,13,14,15,16]. In contrast, the symptoms of posterior circulation ischemic stroke are unapparent and complex [17], and the NIHSS has the problem of severely underestimating the degree of stroke in patients; there are even patients with posterior circulation ischemic stroke that have a score of 0 [18,19]. Furthermore, there is the uncertainty of the onset of stroke symptoms, which makes it more crucial to be able to more accurately assess the severity and prognosis of patients with imaging data. However, no automated diagnostic system has been developed for posterior circulation ischemic stroke, and only the semiquantitative pc-ASPECTS can be calculated visually by physicians.

Although it is easy for specialized clinicians to identify the structures of the posterior circulation, it is not possible to spend time quantifying the size and volume of each structure in acute patients. The system developed in this study can effectively quantify each structure of the posterior circulation and the ischemic lesion diagnosed by the physician, and automatically calculate the patient’s quantitative integrated score, significantly reducing the time required by physicians to obtain accurate quantitative assessment information. In the emergency scene, for patients with neurological symptoms, performing noncontrast CT is a necessary examination. The artificial intelligence algorithm can quickly circle out important anatomical structures. Clinicians only need to mark the location of the lesion. A quantitative integrated score can be produced. On the basis of this value, the physician can discuss with the patient and his family members to formulate treatment strategies. For stroke, the location and size of the lesion directly affect the prognosis. Despite the fact that more information collection may increase a little predictive ability, it increases the barriers of application.

To the best of our knowledge, this study presents the first noncontrast CT-based quantitative automated assessment tool. If the quantitative integrated score can accumulate samples for different treatment methods (including endovascular thrombectomy (EVT), mechanical thrombectomy (MT), recombinant tissue plasminogen activator (rt-PA), etc.), then a range of the quantitative integrated score can be proposed for each treatment intervention that promises a good prognosis, and treatment guidelines for posterior circulation ischemic stroke can thus be developed. The current semiquantitative assessment method, the pc-ASPECTS, is based on computed tomography angiography (CTA), perfusion CT (CTP), and MRI images when assessing the prognosis of patients undergoing various vascular in-transit treatments [20,21,22,23,24]. However, noncontrast CT images cannot accurately assess patient prognosis, or even predict prognosis [25].

Because the proportion of posterior circulation ischemic stroke in stroke patients is low, and because there were fewer people completing both CT and MRI examinations, the sample size in this study is limited. Thus, we used image amplification technology to increase the number of images that could be analyzed. Moreover, with less than 10 patients for each treatment method, it is not possible to provide a prognostic quantitative integrated score range for each treatment method, and precise quantitative information of the lesions still relies on the diagnosis of the neurologist. In addition, the modified pc-ASPECTS has an AUC of 0.74 for prognostic prediction, which is suboptimal for clinical purposes. Therefore, more research is needed to increase the model’s performance in identifying ischemic lesions, and to assist clinicians with treatment decisions in cases of posterior circulation ischemic stroke.

## 5. Conclusions

This retrospective study is the first to build a noncontrast CT-based quantitative integrated score tool using the deep learning model, which significantly shortens the evaluation time of quantitative information from 30 min to 1 min, effectively providing clinicians with a reference to assess the prognosis of posterior circulation ischemic stroke. It provides better prognostic value than semiquantitative scores and can serve as a prognostic prediction tool that provides better guidance for clinical decision making. However, the findings of this study need further independent validation on a larger cohort of patients.

## Figures and Tables

**Table 1:** Basic demographic distribution.

	Good Prognosis (Visits = 18)	Poor Prognosis (Visits = 18)	*p*-Value
Gender (%)			0.044 *
Male	13 (72.2%)	7 (38.9%)	
Female	5 (27.8%)	11 (61.1%)	
Age (mean ± SD)	66.83 ± 11.45	74.83 ± 10.09	0.007 *
BMI, kg/m^2^	24.58 ± 4.84	24.23 ± 2.59	0.874
Treatment			1.000
Drugs	16 (88.9%)	15 (83.3%)	
EVT + rt-PA	2 (11.1%)	3 (16.7%)	
Systolic blood pressure, mmHg	148.41 ± 32.79	147.06 ± 29.08	0.959
Diastolic blood pressure, mmHg	82.35 ± 18.69	79.88 ± 14.56	0.890
Heart rate, beats per minute	86.06 ± 16.39	94.44 ± 28.44	0.408
pc-ASPECTS	9.28 ± 0.75	8.78 ± 1.11	0.164
NIHSS	3.00 ± 1.90	7.75 ± 5.39	0.001 *

Good prognosis: mRS ≤ 2; poor prognosis: mRS ≥ 3; * *p*-value < 0.05.

**Table 2:** Performance of the circulation structure after image segmentation model recognition.

Structure	Mean IoU	Subset I IoU	Subset II IoU	Subset III IoU	Subset IV IoU	Subset V IoU
Left lateral cerebellum	0.78	0.77	0.81	0.79	0.75	0.78
Right lateral cerebellum	0.79	0.78	0.83	0.81	0.77	0.76
Left lateral occipital lobe	0.74	0.78	0.73	0.77	0.75	0.67
Right lateral occipital lobe	0.68	0.66	0.74	0.72	0.63	0.65
Left lateral thalamus	0.73	0.72	0.68	0.76	0.79	0.70
Right lateral thalamus	0.75	0.73	0.78	0.79	0.71	0.74
Medulla oblongata	0.82	0.85	0.82	0.84	0.81	0.78
Midbrain	0.83	0.82	0.80	0.86	0.85	0.82
Pons	0.75	0.74	0.77	0.79	0.72	0.73

The intersection over union (IoU): The similarity between annotated pixels and predicted pixels, defined as the size of the intersection divided by the size of the union of the test sets.

## Data Availability

Not available.

## Supplementary Materials

The following supporting information can be downloaded at: https://www.mdpi.com/article/10.3390/jpm12020138/s1, Table S1. Gender stratification basic demographic distribution; Table S2. Age stratification basic demographic distribution.
