# Supplementary material for: Novel and Efficient Quantitative Posterior-Circulation-Structure-Based Scale via Noncontrast CT to Predict Ischemic Stroke Prognosis: A Retrospective Study
Source: J Pers Med. 2022 Jan 20;12(2):138. doi: 10.3390/jpm12020138 (PMC8876281; doi:10.3390/jpm12020138)
Supplement: Supplementary file 1 [file jpm-12-00138-s001.zip › jpm-1428480-supplementary.pdf]

Table S1. Gender stratification basic demographic distribution.

	**Male**			**Female**		
	**Good prognosis (visits = 13)**	**Poor prognosis (visits = 7)**	***p*-value**	**Good prognosis (visits = 5)**	**Poor prognosis (visits = 11)**	***p*-value**
Age (mean ± SD)	66.00 ± 12.50	72.71 ± 12.32	0.112	69.00 ± 9.00	76.18 ± 8.76	0.110
BMI, kg/m^2	25.00 ± 5.55	24.78 ± 1.10	0.662	22.07 ± 2.53	24.19 ± 3.26	0.061
Treatment			1.000			1.000
Drugs	11 (84.6%)	6 (85.7%)		5 (100.0%)	9 (81.8%)	
EVT + rt-PA	2 (15.4%)	1 (14.3%)		0 (0.0%)	2 (18.2%)	
SBP	146.08 ± 33.94	158.50 ± 18.87	0.300	154.00 ± 32.83	140.82 ± 32.46	0.335
DBP	84.42 ± 20.19	81.00 ± 10.12	0.511	77.40 ± 15.27	79.27 ± 16.94	0.777
HR	86.42 ± 18.08	91.57 ± 11.07	0.419	85.20 ± 13.20	96.27 ± 35.94	0.910
pc-ASPECTS	9.23 ± 0.83	8.43 ± 1.40	0.180	9.40 ± 0.55	9.00 ± 0.89	0.399

Good prognosis: mRS ≤ 2, poor prognosis: mRS ≥ 3

Table S2. Age stratification basic demographic distribution.

	≤70			>70		
	**Good prognosis (visits = 13)**	**Poor prognosis (visits = 7)**	***p*-value**	**Good prognosis (visits = 5)**	**Poor prognosis (visits = 11)**	***p*-value**
Gender (%)			0.383			0.266
Male	10 (71.4%)	4 (44.4%)		3 (75.0%)	3 (33.3%)	
Female	4 (28.6%)	5 (55.6%)		1 (25.0%)	6 (66.7%)	
BMI, kg/m^2	24.94±5.25	24.17 ± 1.72	0.975	21.55 ± 3.25	24.67±3.36	0.105
Treatment			1.000			1.000
Drugs	12 (85.7%)	8 (88.9%)		4 (100.0%)	7 (77.8%)	
EVT + rt-PA	2 (14.3%)	1 (11.1%)		0 (0.0%)	2 (22.2%)	
SBP	152.54 ± 35.47	160.25 ± 32.45	0.716	135.00 ± 19.75	135.33 ± 21.00	1.000
DBP	84.46 ± 18.95	82.25 ± 17.32	0.856	75.50 ± 18.57	77.78 ± 12.30	0.587
HR	87.69 ± 18.28	83.00 ± 11.78	0.545	80.75 ± 6.85	105.89 ± 35.85	0.037*
pc-ASPECTS	9.21 ± 0.70	8.56 ± 1.24	0.169	9.50 ± 1.00	9.00 ± 1.00	0.386

Good prognosis: mRS ≤ 2, poor prognosis: mRS ≥ 3; * p-value < 0.05.
